# Indocyanine green angiography-guided thyroidectomy versus conventional thyroidectomy for preserving parathyroid function: study protocol for a randomized single-blind controlled trial

**DOI:** 10.3389/fendo.2023.1193900

**Published:** 2023-05-08

**Authors:** Pablo Moreno-Llorente, Guillermo García-González, Mireia Pascua-Solé, Arantxa García-Barrasa, Sebastián Videla, José Luis Muñoz-de-Nova, the GuiArte Study Group

**Affiliations:** ^1^Unit of Endocrine Surgery, Department of Surgery, Hospital Universitari de Bellvitge, Universitat de Barcelona, L’Hospitalet de Llobregat, Barcelona, Spain; ^2^Clinical Research Support Unit (HUB), Institut d’Investigació Biomèdica de Bellvitge (IDIBELL) (HUB-IDIBELL), Hospital Universitari de Bellvitge, L’Hospitalet de Llobregat, Barcelona, Spain; ^3^Pharmacology Unit, Department of Pathology and Experimental Therapeutics, Faculty of Medicine, University of Barcelona, L’Hospitalet de Llobregat, Barcelona, Spain; ^4^Department of General and Digestive Surgery, Hospital Universitario de La Princesa, Instituto de Investigación Sanitaria Princesa (IIS-IP), Universidad Autónoma de Madrid (UAM), Madrid, Spain

**Keywords:** indocyanine green, angiography, total thyroidectomy, parathyroid glands, permanent hypoparathyroidism, vascular feeding pattern

## Abstract

**Introduction:**

Angiography with indocyanine green (ICG) fluorescence performed before thyroidectomy would allow identification of the vascularization of parathyroid glands, maximizing efforts for preserving functioning glands intraoperatively. The rationale of the study was based on the hypothesis that showing the vascular pattern of the parathyroid glands by means of ICG angiography before performing the thyroidectomy could prevent permanent hypoparathyroidism.

**Methods and analysis:**

We propose a randomized single-blind controlled and multicenter clinical trial to assess the efficacy and safety of ICG angiography-guided thyroidectomy to identify the vascular pattern of the parathyroid glands versus conventional thyroidectomy in patients scheduled for elective total thyroidectomy. Patients will be randomized 1:1 to ICG angiography-guided thyroidectomy (experimental group) or conventional thyroidectomy (control group). Patients in the experimental group will undergo ICG angiography before thyroidectomy to identify the feeding vessels of the parathyroid glands and then, post-thyroidectomy ICG angiography to predict immediate parathyroid gland function by scoring the degree of fluorescence of the glands. Patients in the control group will undergo post-thyroidectomy ICG angiography only. The primary outcome measure will be the rate of patients with permanent hypoparathyroidism. Secondary outcome measures will be rate of postoperative hypoparathyroidism, the percentage of well vascularized parathyroid glands remaining in situ, the levels of iPTH and serum calcium after surgery and the influence of the type of vascular pattern of the parathyroid glands over these outcomes, as well as the safety profile of ICG angiography.

**Discussion:**

The results will contribute to adopt a new surgical strategy based on intraoperative ICG angiography before performing total thyroidectomy, according to which the rate of permanent hypoparathyroidism could be substantially reduced.

**Clinical trial registration:**

ClinicalTrials.gov. identifier NCT05573828.

## 1 Introduction

Permanent hypoparathyroidism as a sequela of thyroid gland surgery continues to be a real and challenging clinical problem. Parathyroid failure may occur after surgical procedures to treat both benign and malignant thyroid disorders usually as a result of trauma, devascularization, parathyroid autotransplantation, or inadvertent resection of the glands (1). Most people with permanent hypoparathyroidism have to take calcium and vitamin D supplements for life, with long-term consequences affecting the quality of life and increasing economic costs (2, 3). The reported rate of permanent hypoparathyroidism is highly variable between less than 4% and up to 15% depending on the different definition of hypoparathyroidism used in each study and especially to the different time cutoff applied to distinguish transient from permanent hypoparathyroidism (4–6).

Novel fluorescence techniques have been developed for improving intraoperative identification of the parathyroid glands and to avoid postoperative hypocalcemia. Indocyanine green (ICG) angiography has been shown to be a valuable technique for identifying and assessing the perfusion of the parathyroid glands during total thyroidectomy (7, 8). Quantitative scoring systems based on a black and white scale depending on the amount of ICG flowing through the gland and categorized as 0, black (nonvascularized), 1, gray/heterogeneous (partially vascularized), and 2, white (well vascularized) have been successfully used for predicting early postoperative hypocalcemia after total thyroidectomy (9, 10). Moreover, a single-gland ICG score of 2 in any parathyroid gland after completion of thyroid surgery is a reliable method in prediction of early post-thyroidectomy hypocalcemia (11, 12). Recently, Benmiloud et al. (13) have shown the feasibility of using intraoperative ICG mapping angiograms of the parathyroid glands to improve vascular preservation during thyroid surgery. In an assessment of 76 parathyroid glands, those glands with informative angiography (visible vascular pattern) showed a higher percentage of ICG score of 2 than for parathyroid glands with uninformative angiography (13).

In a previous study of our group, a comparison of immediate and permanent hypocalcemia was made in two cohorts of patients who underwent total thyroidectomy and ICG-angiography (14). In patients included in the prospective cohort (n = 36), ICG-angiography was performed after identification of the parathyroid glands to show their vascular supply prior to surgical removal of the thyroid gland (angiography-guided thyroidectomy), whereas in the historical comparative cohort (n = 84), ICG-angiography was performed only at the end of the surgery. The rates of early and permanent hypocalcemia were significantly lower in the angiography-guided thyroidectomy group (5.6% *vs.* 26.2%, *p* = 0.011, and 0% *vs.* 11.9%, *p* =0.032, respectively) than in controls. Moreover, a significant higher rate of well vascularized parathyroids at the end of the surgery (score 2) in the angiography-guided thyroidectomy group (52.9% vs. 39.2%, *p* = 0.018) was also seen (14). Results obtained in this cohort study provided the rationale for the design of a randomized controlled clinical trial. The hypothesis is that showing the vascular map of the parathyroid glands before performing the thyroidectomy by means of ICG angiography could prevent the development of postoperative hypoparathyroidism. Therefore, we here describe the protocol of a randomized, single-blind, controlled, and multicenter study to assess the efficacy and safety of ICG angiography-guided thyroidectomy versus conventional thyroidectomy in patients undergoing total thyroidectomy.

## 2 Materials and methods

### 2.1 Research design

**Figure 1:**
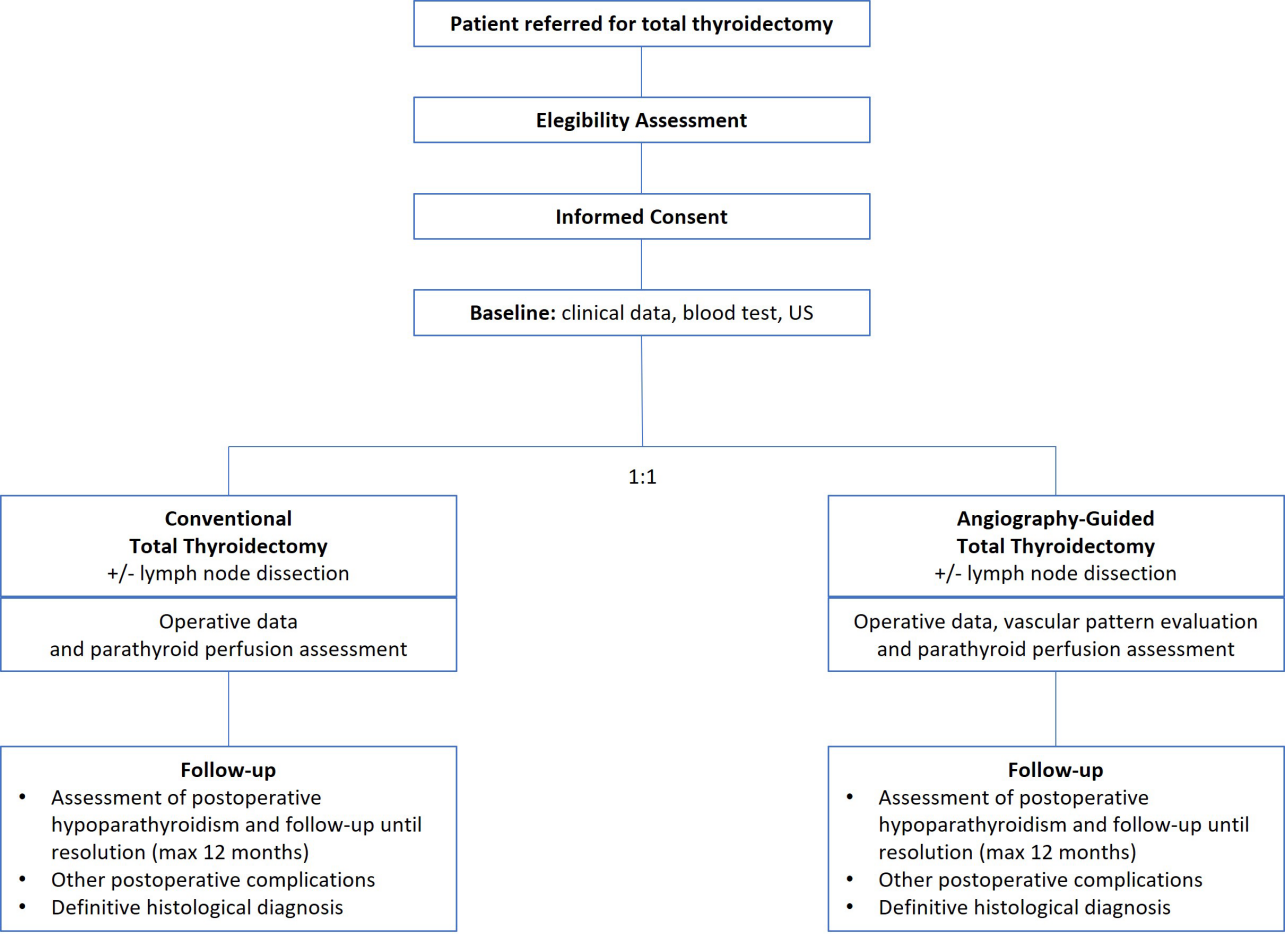
Flow chart of the GuiArte trial.

This study is a randomized, single-blind, controlled, parallel arm, and multicenter trial. In this single-blind design only the patient is blind to the allocation. Recruitment starts in October 2022 and ends in December 2024. Participants will be randomly (1:1) divided into ICG angiography-guided thyroidectomy (experimental group) that included an ICG-angiography before thyroidectomy to identify the vascular pattern of the thyroid glands, and conventional thyroidectomy (control group). Post-thyroidectomy ICG to predict parathyroid function by scoring the degree of fluorescence of the glands will be performed in all patients. The study is registered in the ClinicalTrials.gov (NCT05573828) and will follow the guidelines of Consolidated Standards for Reporting Trials (CONSORT) (15). Flow chart is shown in **Figure 1**.

### 2.2 Research setting

This study will recruit 394 eligible subjects from the Units of Endocrine Surgery of tertiary care hospitals in Spain. The University Hospital of Bellvitge in Barcelona (Spain) will be the reference center. Inclusion and exclusion criteria are shown in **Table 1**.

**Table 1:** Inclusion and exclusion criteria.

**1.- Inclusion Criteria** (1) Patients ≥ 18 years of age with a surgical indication for elective total thyroidectomy with or without central cervical lymph node dissection due to thyroid pathology. (2) The patient has the capacity to understand the study and agrees to participate in it, signing the corresponding informed consent document.
**2.- Exclusion Criteria** (1) Previous surgical intervention on the thyroid or parathyroid gland. (2) Associated hyperparathyroidism that requires associating a parathyroidectomy in the same surgical act. (3) Patients with contraindications for the administration of ICG except for patients with clinical hyperthyroidism, autonomous thyroid adenomas, and focal and diffuse changes of the thyroid gland who will undergo total thyroidectomy. (4) Current drug use or alcohol abuse that could interfere with compliance with the study requirements. (5) Participation in any other drug trials in the month prior to randomization.

### 2.3 Randomization and blinding

After baseline assessment, the study will use a 1:1 ratio to randomly divide the subjects into 2 groups. Randomization will be performed using a computer-generated table of random numbers. The group assignment will be performed by an independent statistician who would be blinded to the recruitment, intervention, and evaluation of participants. Blinding of researchers (who perform the surgical procedures) to group assignment would not be possible to the specific nature of the study. The principal investigator at each participating center will be aware of the randomization arm before entering the operating room. However, statisticians who will perform the statistical analysis after the follow-up of patients will be blinded to group assignment. At the same time, the study is single-blind randomized trial. In this study, all participants will be unaware of the allocation to the experimental or the control group.

### 2.4 Intervention

All patients will be managed similarly in the preoperative period. Supplementation with vitamin D will be recommended for patients with 25-OH vitamin D deficit (< 20 ng/mL). None of the patients will receive preoperative calcium supplementation. The protocol for standard thyroidectomy includes starting with luxation of each thyroid lobe and performing careful dissection to minimize damage to the parathyroid glands, followed by search and visual identification of the glands in each lobe, as well as in orthotopic localizations.

#### 2.4.1 ICG angiography-guided thyroidectomy

All the participant surgeons will conduct a formative meeting with the leading group to homogenize the procedures and the definitions. The procedure will include performing an ICG angiography of the parathyroid glands before removal of the thyroid gland. To this purpose, after the luxation of the thyroid lobe and the identification of the parathyroid glands, 1 mL of the contrast material will be administered through a peripheral vein after dilution of a powdered vial of 25 mg (Verdye^®^, Diagnostic Green GmbH, Aschheim-Dornach, Germany) of ICG in 10 mL of sterile water. After a few seconds, ICG-enhanced fluorescence imaging of the vascular pattern of the parathyroid glands previously identified will be acquired using a near-infrared camera. Visualization of the vascular pattern of the parathyroids will include identification of a clear feeding vessel with its pedicle (defined pattern) or a vascular network around the parathyroid gland without a clear feeding vessel (non-defined pattern). After identification of the vascular map of each parathyroid gland, the dissection will be performed in a guided manner to minimize its damage. The surgical procedure will continue with total removal of the thyroid lobe and then performing an ICG angiography of the parathyroids after injection of 3 mL of the contrast material, and obtaining a black and white near-infrared view. The fluorescence of the glands (back and white imaging) will be assessed intraoperatively by the operating surgeon, and the degree of fluorescence will be classified according to the color of the gland reflecting the degree of perfusion. The color of the gland can vary from black (suggesting that it is not vascularized and likely non-viable), to white (suggesting that it is well vascularized and viable). Glands will be classified as 0, black (nonvascularized); 1, gray/heterogeneous (partially vascularized); and 2, white (well vascularized). Both autotransplanted and parathyroid glands identified in the histopathological report are devascularized and will be scored as 0. The procedure will be the same in the other lobe, but the initial doses will be 3 mL. During the dissection, repeated doses to reevaluate the vascularization are allowed when needed until a maximum of 5 mg/kg. In patients undergoing central neck dissection, ICG angiography to assess the degree of parathyroid glands vascularization will be performed after each procedure, lymphadenectomy and thyroidectomy.

#### 2.4.2 Conventional thyroidectomy

Conventional thyroidectomy will include identification of the parathyroid glands visually (naked eye) and performing a total thyroidectomy followed by an ICG angiography of the parathyroids (as described in the angiography-guided thyroidectomy), assessing the fluorescence the glands and scoring the color reflecting the degree of vascularization as 0, 1, or 2. In patients undergoing central neck dissection, ICG angiography will be performed also after each procedure, lymphadenectomy and thyroidectomy.

In both ICG angiography-guided and conventional thyroidectomy, the case report form will be completed at real time at the end of surgery.

### 2.5 Postoperative care and follow-up

During hospitalization, the clinical course of patients will be controlled to assess the presence of hypoparathyroidism, complications, or adverse events (16). Intact parathyroid hormone (iPTH) levels will be measured at 24 hours postoperatively (i.e. the morning after surgery) according to the technique used at each hospital laboratory. Corrected serum calcium levels will be measured 24 hours after surgery. Clinical practice guidelines of each center will be followed for postoperative administration of calcium and vitamin D. Those patients with postoperative hypoparathyroidism will be followed clinically in association with periodical measurements of iPTH, corrected serum calcium, and vitamin D levels at 1, 3, 6, and 12 months after surgery or until hypoparathyroidism resolution.

### 2.6 Definitions

Postoperative hypoparathyroidism will be defined by the appearance of symptoms of hypocalcemia after surgery, presence of hypocalcemia (corrected serum calcium levels < 8 mg/dL) in the absence of symptoms, and/or requirement of calcium and/or vitamin D supplementation before this measurement. Permanent hypoparathyroidism will be defined as the need of treatment with calcium and/or vitamin D to maintain corrected serum calcium levels in the normal range or to prevent symptoms of hypocalcemia 12 months after surgery.

An adverse event (AE) will be defined as a medical occurrence in a patient participating in the study, regardless of the causal relationship to the treatment assigned.

### 2.7 Outcome assessment

#### 2.7.1 Primary outcome

To analyze if those patients included in the experimental group have a lower rate of permanent hypoparathyroidism compared with the control group.

#### 2.7.2 Secondary outcomes

(1) To compare the rate of postoperative hypoparathyroidism between both study groups.
(2) To compare the number of well-vascularized parathyroid glands (ICG score of 2) remaining *in situ* after thyroidectomy between both groups.
(3) To compare the serum levels of iPTH and corrected calcium 24 hours after surgery between both groups.
(4) To compare the duration of surgery as the time interval between the start of skin incision and its final closure between both groups.
(5) To analyze the influence of the type of vascular pattern (defined or undefined) of the parathyroid glands in angiography-guided thyroidectomy group in the outcomes.
(6) To describe the safety profile and adverse events of the ICG angiography.

### 2.8 Sample size and statistical analysis

In a previous single-center study, it was found that in a prospective cohort of patients in which ICG arteriography was performed before removal of the thyroid gland, the rate of permanent hypoparathyroidism was 0% as compared with 11.9% in a cohort of historical controls (14). Based on a more conservative approach, a rate of permanent hypoparathyroidism of 2% is estimated in the ICG angiography-guided thyroidectomy and 8% in the control group. Assuming 1:1 allocation, an alpha error of 5%, a statistical power of 80%, and a loss rate of approximately 5% in each group, 197 patients per arm will be needed. Therefore, a total of 394 patients will be recruited. It is estimated that these patients will be included in the study between January 2023 and December 2024.

Study variables will be collected by the researchers through clinical visits and results of laboratory analyses. Data will be stored in a codified electronic database specifically designed for the purpose of the study. Monitorization of study data will be performed by external personnel unaware of details of the study. To examine the benefits of identifying the vascular pattern of the parathyroid glands using an ICG angiography before thyroidectomy for preventing permanent hypoparathyroidism, a full analysis set will be performed, that is, all patients randomized who will be operated on, and followed postoperatively until permanent hypoparathyroidism will be resolved or for a maximum of 12 months after surgery. Categorical variables will be expressed as frequencies and percentages, and continuous variables as mean ± standard deviation or median and interquartile range (IQR) (25th-75th percentile). The Shapiro-Wilks test will be used to assess the normal distribution of data and the Levene’s test to assess homoscedasticity. Categorical variables will be analyzed with the chi-square test or the Fisher’s exact test, and quantitative variables with the Student’s *t* test or the Mann-Whitney *U* test according to conditions of application. The risk ratio (RR) and the 95% confidence interval (95%) will be calculated to assess the probability of permanent hypoparathyroidism. Univariate and multivariate logistic regression models adjusted by confounding variables will be performed to identify risk factors for permanent hypoparathyroidism. *P* < 0.05 is set as the significance level. The Statistical Package for the Social Sciences (SPSS) version 26 for Windows will be used for data analysis.

## 3 Discussion

Permanent hypoparathyroidism, a feared complication of thyroidectomy, in particular after total thyroidectomy for cancer, leads to substantial morbidity making the patient dependent on replacement therapy for life (1). Therefore, the feasibility of a surgical innovation technique allowing to preserve “in situ” more well perfused parathyroid glands during thyroidectomy and, consequently, contributing to prevent postoperative hypoparathyroidism is a clinically relevant contribution. The use of an ICG angiography before removal of the thyroid gland for intraoperative identification of the vascular supply of the parathyroids involves a change in the way thyroidectomy is performed. Previous experience with the use of ICG angiography has been focused on assessing the degree of fluorescence based on a black and white scoring system as a reflection of the degree of vascularization of the glands. Angiography-guided thyroidectomy is a further step to identify the parathyroid glands by means of visualizing the feeding vasculature of each individual gland. Moreover, ICG angiography is an easy procedure that does not add much time to the operation or include complicated or difficult maneuvers given that preparation and injection of the fluorescent dye is very simple. The main expected results of this study are a lower rate of permanent hypoparathyroidism, and a higher number of well-vascularized parathyroid glands left *in situ* in the angiography-guided thyroidectomy as compared to conventional thyroidectomy, with statistically significant differences in the comparison of these variables between the study groups.

These results will provide robust evidence of the advantages of incorporating pre-thyroidectomy ICG angiographic detection of the vascular mapping of the parathyroid glands for preventing permanent hypoparathyroidism. Accordingly, angiography-guided thyroidectomy may be a recommended technical procedure for patients undergoing total thyroidectomy.

## Ethics statement

The study has been approved by the Clinical Research Ethics Committee of Hospital Universitari de Bellvitge (approval code PR0042/22). The patients/participants provided their written informed consent to participate in this study.

## Author contributions

PM-L and JM-d-N designed the study and developed the protocol. GG-G, MP-S and AG-B helped in the development of the protocol. JM-d-N and GG-G helped in sample size calculation and statistical analysis plan. PM-L and JM-d-N wrote the manuscript. GG-G, MP-S and AG-B critically reviewed the manuscript. All authors contributed to the article and approved the submitted version.
